# External Quality Assessment 2.0: The Importance of a Standardized Implementation of TILs for Daily and Trial Practices

**DOI:** 10.3390/cancers14153762

**Published:** 2022-08-02

**Authors:** Inne Nauwelaers, Nele Laudus, Dieter Peeters, Balazs Acs, Carsten Denkert, Stefan Michiels, Hugo Horlings, Kalliopi P. Siziopikou, Scott Ely, Dimitrios Zardavas, Roberts Mustimbo, John Bartlett, Giuseppe Floris, Johan Hartman, Carolien H. M. van Deurzen, Dorien Ceusters, Els Dequeker, Roberto Salgado

**Affiliations:** 1 Biomedical Quality Assurance Research Unit, Department of Public Health and Primary Care, University of Leuven, Kapucijnenvoer 35d, 3000 Leuven, Belgium; inne.nauwelaers@kuleuven.be (I.N.); nele.laudus@kuleuven.be (N.L.); dorien.ceusters@kuleuven.be (D.C.); els.dequeker@kuleuven.be (E.D.); 2 CellCarta NV, 2610 Antwerp, Belgium; dieter-peeters@telenet.be; 3 Department of Pathology, AZ Sint-Maarten, 2800 Mechelen, Belgium; 4 Department of Oncology and Pathology, Cancer Center Karolinska, Karolinska Institute, 171 64 Stockholm, Sweden; balazs.acs@ki.se (B.A.); johan.hartman@ki.se (J.H.); 5 Department of Clinical Pathology and Cancer Diagnostics, Karolinska University Hospital, 171 76 Stockholm, Sweden; 6 Institute of Pathology, Philipps-University Marburg and University Hospital Marburg (UKGM), Baldingerstr. 1, 35043 Marburg, Germany; carsten.denkert@uni-marburg.de; 7 Department of Biostatistics and Epidemiology, Gustave Roussy, University Paris-Saclay, 94800 Villejuif, France; stefan.michiels@gustaveroussy.fr; 8 Oncostat U1018, Inserm, Labeled Ligue Contre le Cancer, University Paris-Saclay, 94800 Villejuif, France; 9 Division of Molecular Pathology, The Netherlands Cancer Institute, 1066 CX Amsterdam, The Netherlands; h.horlings@nki.nl; 10 Department of Pathology, The Netherlands Cancer Institute, 1066 CX Amsterdam, The Netherlands; 11 Department of Pathology, Section of Breast Pathology, Northwestern University, Chicago, IL 60611, USA; ksiziopi@nm.org; 12 Translational Medicine, Bristol-Myers Squibb, Princeton, NJ 08540, USA; scott.ely@bms.com (S.E.); mustimbo.roberts@bms.com (R.M.); 13 Oncology Clinical Development, Bristol-Myers Squibb, Princeton, NJ 08540, USA; dimitrios.zardavas@bms.com; 14 Edinburgh Cancer Research Centre, Institute of Genetics and Molecular Medicine, Edinburgh EH4 2XR, UK; john.bartlett@ed.ac.uk; 15 Laboratory of Translational Cell & Tissue Research, Department of Imaging and Pathology, University of Leuven, 3000 Leuven, Belgium; giuseppe.floris@uzleuven.be; 16 Department of Pathology, University Hospitals Leuven, 3000 Leuven, Belgium; 17 Department of Pathology, Erasmus Medical Center-Cancer Institute, 3015 GD Rotterdam, The Netherlands; c.h.m.vandeurzen@erasmusmc.nl; 18 Department of Pathology, GZA-ZNA Hospitals, 2610 Antwerp, Belgium; 19 Division of Research, Peter Mac Callum Cancer Centre, Melbourne, VIC 300, Australia

## Abstract

New assays are developed regularly to improve health care for patients. It is important to ensure that assays are performed correctly. Therefore, it is advised to participate in training and proficiency (competence assessment) programs. Tumor infiltrating lymphocytes (TILs) might improve the estimates of response to therapy and prognosis. Herewith, we propose a new training and proficiency program in which each pathologist can train and test themselves regarding TILs (and PDL1) scoring.

Increasing data suggests that an intact immune system is required for improved outcomes in patients with Human Epidermal Growth Factor Receptor 2 (HER2+) and Triple Negative Breast Cancer (TNBC). Tumor Infiltrating Lymphocytes (TILs), an indicator of anti-tumor immune response in the host, are an established prognostic factor [1], and are predictive for response to immunotherapy in TNBC [2]. An accurate and reproducible assessment of TILs is therefore necessary for daily and clinical trial practices.

TILs are currently measured by visual evaluation of hematoxylin-eosin stained (H&E) slides. In addition, there are ongoing efforts for assessment of TILs by machine learning and artificial intelligence-methods [3]. The International Immuno-Oncology Biomarker Working Group (also called the TILs-WG, https://www.tilsinbreastcancer.org/ (accessed on 15 July 2021)) has developed an easy and accessible method to assess TILs using H&E slides [4]. The TILs-WG has also developed a freely available self-training tool, including audio-visual materials (https://www.youtube.com/watch?v=aPa-pXIBBlU (accessed on 7 January 2021)) and has completed several inter-laboratory comparison studies, i.e., ring studies that showed this method to be reproducible [5]. These TIL training materials are freely available on the WG’s website.

The assessment of TILs is increasingly being introduced in daily practices worldwide [4,6]. Hence, high-quality training and continuous competence assessment is crucial for sustained implementation of this biomarker in thousands of pathology laboratories worldwide.

Standardized and reproducible assay results are key to successful biomarker implementation in clinical practice. Inaccurate diagnostic results can have a potential negative impact in patient clinical management. An increasing number of governmental and regulatory bodies in many countries recognize the importance of requiring a minimum level of quality management in clinical laboratories [7]. New diagnostic methods and increasing awareness about assay errors and their consequences emphasize the great importance of quality in health care settings [8]. A laboratory quality management system (QMS), such as compliance with ISO15189 [9] or through the College of American Pathologists (CAP) is a step forward to ensure quality of testing [10].

This ISO-standard, for example, states that “interlaboratory comparison program(s) chosen by the laboratory shall, as far as possible, provide clinically relevant challenges that mimic patient samples […]” [9]. As recognized by the ISO-standard, participation in external quality assessment (EQA) schemes, also called proficiency testing, allows monitoring of laboratory performance, comparisons between laboratories and provides feedback to ensure consistent delivery of services over time. When partaking in EQA schemes organized by, for example, NORDIQC, UK NEQAS, CBQA, etc., participants receive a set of samples they must analyze according to their routine protocols. All participants receive samples from the same source, so results can be compared. After submission of results, participants receive feedback on their performance, as well as information about their performance compared to other participants. This practice arms laboratories/pathologists with knowledge to increase their performance and increases confidence to patients and clinicians in the results provided by the laboratory.

EQA schemes are an essential tool when implementing new types of assessments of biomarkers [11], and participation in EQAs can result in increased performance and better standardization across different laboratories worldwide [12,13]. However, in many parts of the world, certainly in low-to-middle-income resource countries, implementing, for example, an ISO15189-standard in pathology laboratories is not always feasible. Nevertheless, even in these settings, participation in EQAs can be helpful, certainly when the “assay” is the pathologist evaluating a morphological biomarker on an H&E-slide, as is the case with TILs.

A team of experts from the TILs-WG, in collaboration with the Biomedical Quality Assurance Research Unit of the University of Leuven, have set up a worldwide, freely available training- and EQA-scheme using an new developed online platform, to train and investigate the competence of pathologists in evaluating TILs as a new morphologic biomarker in TNBC, and, as PD-L1-assays in breast cancer mostly stain TILs, also PD-L1 (Figure 1; TILs and PD-L1 Training Course: International TILS Working Group (tilsinbreastcancer.org, accessed on 15 July 2021)). The overall purpose of this study is to support pathologists implementing TILs scoring in clinical practice as a prognostic biomarker for TILs, and as a predictive combined TILs/PD-L1-biomarker, as both are associated with improved outcomes in TNBC. For example, in IMpassion130, a phase III clinical trial that assessed nab-paclitaxel with Atezolizumab or placebo for patients with newly diagnosed inoperable and/or metastatic TNBC, the Hazard Ratio (HR) of benefit to immunotherapy is similar for PD-L1 IC+/any TILs (0.65 for PFS and 0.71 for OS), compared to the HR for any PD-L1 IC+ and TILs > 10% (0.64 for PFS and 0.75 for OS), suggesting that both are complementary metrics [14].

TILs are thus increasingly considered to be complementary to PD-L1. Since the immune cell staining is an important component of PD-L1 assays in breast cancer immunotherapy trials, a combined assessment of TILs and PD-L1 might prove to be a successful complementary approach to use in daily practice, the possibility of which will be studied in the EQA scheme mentioned above. In some laboratories, mostly in low-to-middle-income countries, TILs are pre-screened as a cost saving measure, since, if no immune cells are present, any PD-L1-assay assessing the expression of PD-L1 on immune cells will likely be negative [15]. Specifically, for a given TIL percentage, we question how likely is it that the PD-L1 IC score is above the clinically relevant cut-off point.

The EQA scheme introduced here will be organized on an individual level, rather than on a laboratory level, so every pathologist that wants to train themselves can participate, with no previous experience necessary. The scheme will contain multiple modules (Figure 1). After registration on the digital platform (TILS Login Page (agoko.be, accessed on 19 October 2021)), a training module for TILs estimation will be available (e.g., theoretical explanations, comparative exercises, videos, etc.). Another module will probe the pathologists experience and levels of training on TIL estimation as well as the current use of PD-L1 and TILs in clinical practice. A subsequent module will contain an EQA where we ask participants to score the TILs and PD-L1 on digital slides. Digital slides, made in a single center, will be made available. The PD-L1 stained samples were stained with multiple assays (SP142, SP263 and 22C3), all in the same laboratory and according to FDA-approved assay requirements.

After submission of the results, an expert team will analyze the data. If variables are found that influence scoring, there will be a feedback mechanism to the participants. Feedback on the organization and quality of the EQA scheme itself will be collected to learn and improve the platform for future use.

A comparison will be made of PD-L1 scores in different tumor compartments (immune cells and tumor cells, and the combination hereof) to investigate the complementarity of TILs to PD-L1 testing, wherein each variable will be assessed in a continuous manner, including but not limited to the assay-specific scoring-algorithm as developed by the vendor. This will allow us to analyze, in an unbiased manner, the analytical performance of pathologists on TILs and PD-L1, on both tumor cells and immune cells, as well as the variance between pathologists.

Participation in this EQA by pathologists worldwide should be encouraged, as only unbiased EQA-schemes in the scientific community can identify problems and obstacles, and mitigate these issues in further training-modules and subsequent competence assessments. For the sake of our patients, this partnership between academia and industry to perform unbiased EQA and learn from it, is key to the sustained implementation of a biomarker in daily practices worldwide. We need to learn from previous experiences on how biomarkers were introduced and improve the process by which biomarkers become widely adopted in daily practice. This project aims to develop a framework that is in the best interest of our patients [12] and this framework, as proposed by the most important pathology organizations worldwide, should be used for all future biomarker assessments before incorporation in daily practices can be considered.

## Figures and Tables

**Figure 1:**
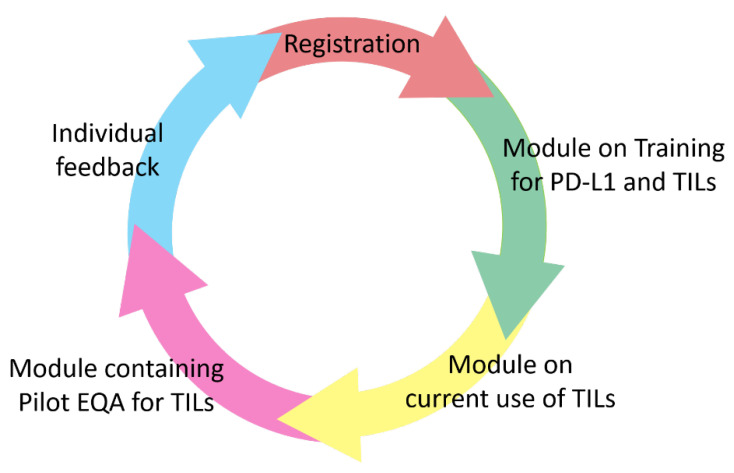
After registration, through www.tilsinbreastcancer.org (accessed on 15 July 2021), a training module will be available for PD-L1 and TILs. Next, a pilot EQA module will be available to assess the current practices and competences of pathologists to score TILs. Finally, individual feedback will be offered to each participant separately.
